# Editorial: Aquatic Pharmacology—Temperature Sensitive Medication

**DOI:** 10.3389/fvets.2021.755585

**Published:** 2021-09-28

**Authors:** Lisa A. Tell, Prapansak Srisapoome, Chi-Chung Chou

**Affiliations:** ^1^Department of Medicine and Epidemiology, School of Veterinary Medicine, University of California, Davis, Davis, CA, United States; ^2^Department of Aquaculture, Faculty of Fisheries, Kasetsart University, Bangkok, Thailand; ^3^Department of Veterinary Medicine, College of Veterinary Medicine, National Chung Hsing University, Taichung, Taiwan

**Keywords:** temperature-sensitive, aquatic, pharmacology, pharmacokinetics, tilapia and rainbow trout

Infectious diseases plague aquaculture species and continue to be ongoing threats. There are very few antimicrobial drugs approved for use in aquatic animals compared to mammals. Therefore, in order to protect the health and welfare of aquatic species and ultimately the health of humans consuming these food products, research investigations and knowledge dissemination in the broader sense of pharmacology for aquatic species are imperative. To our knowledge, out of more than 300 veterinary and aquatic science journals listed in the science citation index (SCI), there are no journals or single sections in any journal that are dedicated specifically to pharmacologic research in aquatic species. Consequently, knowledge advancement in the broad discipline of aquatic pharmacology falls significantly behind that of the land animals. For this reason, this special edition was conceived to create the first collection of its kind.

The specific goal of this special edition was to centralize scientific information regarding the development and application of antimicrobial drugs (including but not limited to antibacterial, antifungal, and antiparasitic agents) used in aquatic animals in the aspects of drug efficacy (both *in vitro* and *in vivo*), safety/toxicity, and pharmacokinetics including bioavailability, distribution, metabolism, and tissue residue depletion. Ideally, this special edition would have also attracted manuscripts addressing alternatives to antimicrobial drug uses, application of other therapeutic agents like antiseptics/disinfectant (for external infection), herbal/algal extracts and their isolated compounds (1), or novel chemicals showing promising results for aquaculture application. The potential diversity of topics underscores the importance for aquaculture species to receive more scientific attention.

This special edition turned out to be comprised of scientific papers focusing mostly on the clinical aspects of aquatic pharmacology and pharmacokinetics, which may reflect the lack of antimicrobial agents to treat aquatic infectious diseases (2) and thus need for more precise instructions for drug use. Two manuscripts studied one of the most renowned features of aquatic species that makes aquatic pharmacokinetics fascinating: temperature-sensitive pharmacokinetics. Temperature-dependent residue depletion of tiamulin in Nile tilapia (*Oreochromis niloticus*) (Cao et al.) and the development of a temperature-related, physiological pharmacokinetic model for enrofloxacin and ciprofloxacin in rainbow trout (Yang et al.) were studied. The calculated withdrawal periods for tiamulin in Nile tilapia were 12 days at 19°C, 9 days at 25°C, and 7 days at 30°C, making 228 an optimal degree-day suggestion for Nile tilapia raised at 19°C and above. On the other hand, the withdrawal intervals for enrofloxacin and ciprofloxacin and at different dosages and water temperatures ranged from 80 to 272 degree-days in rainbow trout. These values were shorter than the European Medicines Agency (EMEA) enforced label withdrawal period (500 degree-days) in fish (3). Overall these two studies highlight the unique feature of the influence of temperature on tissue residue depletion kinetics and the need for a useful model to either predict the optimal dosage regimens (2) or tissue residues under different dosage regimens and water temperatures (Yang et al.).

Conventional pharmacokinetic studies at specific temperatures were also submitted to this special issue, including pharmacokinetic and efficacy study of acyclovir against cyprinid herpesvirus-3 in *Cyprinus carpio* (Carde' et al.), the comparative pharmacokinetics of ketoprofen in Nile tilapia (*Oreochronis niloticus*) and rainbow trout (*Onchorhynchus mykiss*) (Greene et al.), the pharmacokinetics of doxycycline in channel catfish (*Ictalurus punctatus*) (Xu et al.), and the pharmacokinetics of oxytetracycline in the Chinese soft-shell turtle (*Pelodiscus sinensis*). These manuscript submissions encompass antiviral and antibacterial drugs, covering also aquatic species other than fish. These studies provide information pivotal for the appropriate use of these medications in aquaculture and bioavailability data for the development of future formulations. Last but not least, the efficacy of antiparasitic agent emamectin benzoate was orally tested on saltwater-cultured hybrid grouper (*Mycteroperca tigris* × *Epinephelus lanceolatus*) infected with sea lice. It was revealed that individual fish showed significant treatment variation by oral-feeding but drug effectiveness could last up to 14 days after treatment (St-Hilaire et al.), this article received most total views of the six papers published on this special topic, which may reflect the higher demand for effective antiparasitic medicine.

We hope the completion of this special topic edition provides the basis for a future compilation of concepts and findings pertaining to not only pharmacokinetics, but also pharmacodynamics, pharmacotherapeutics, pharmacogenetics, pharmacovigilance, and immunopharmacology in all aquatic species, including those of domestic, wild, and laboratory origins. In addition, immunomodulators, probiotics, vitamins, or other general health-promoting substances that are not aimed for disease treatment could also be considered. The utilization of sedatives/anesthetics for better experimental quality and aquatic animal welfare should be an important addition in the future (4). We believe these diversified pharmacology disciplines strengthen the necessity of a section dedicated to aquatic researches in Frontiers in Veterinary Science.

## Author Contributions

All authors listed have made a substantial, direct and intellectual contribution to the work, and approved it for publication.

## Conflict of Interest

The authors declare that the research was conducted in the absence of any commercial or financial relationships that could be construed as a potential conflict of interest.

## Publisher's Note

All claims expressed in this article are solely those of the authors and do not necessarily represent those of their affiliated organizations, or those of the publisher, the editors and the reviewers. Any product that may be evaluated in this article, or claim that may be made by its manufacturer, is not guaranteed or endorsed by the publisher.
